# Visible Light Communication: A System Perspective—Overview and Challenges

**DOI:** 10.3390/s19051153

**Published:** 2019-03-07

**Authors:** Saeed Ur Rehman, Shakir Ullah, Peter Han Joo Chong, Sira Yongchareon, Dan Komosny

**Affiliations:** 1 Department of Electrical and Electronic Engineering, Auckland University of Technology, Auckland 1010, New Zealand; shakir.ullah@aut.ac.nz (S.U.); peter.chong@aut.ac.nz (P.H.J.C.); 2 Department of Information Technology and Software Engineering, Auckland University of Technology, Auckland 1010, New Zealand; sira.yongchareon@aut.ac.nz; 3 Department of Telecommunications, Brno University of Technology, Technicka 12, 601 90 Brno, Czech Republic; komosny@feec.vutbr.cz

**Keywords:** visible light communication, optical communication, LED communication, VLC networks

## Abstract

Visible light communication (VLC) is a new paradigm that could revolutionise the future of wireless communication. In VLC, information is transmitted through modulating the visible light spectrum (400–700 nm) that is used for illumination. Analytical and experimental work has shown the potential of VLC to provide high-speed data communication with the added advantage of improved energy efficiency and communication security/privacy. VLC is still in the early phase of research. There are fewer review articles published on this topic mostly addressing the physical layer research. Unlike other reviews, this article gives a system prespective of VLC along with the survey on existing literature and potential challenges toward the implementation and integration of VLC.

## 1. Introduction

In the 1980s, the development of high-efficiency red, orange and yellow light emitting diodes (LEDs) have fueled the idea of replacing the solid-state lighting for illumination purpose. It was until 1996 when the first white LED was commercially introduced in the market for sale [1]. LED lights are highly powered efficient, low carbon emissions, free from mercury, durable and produce good quality illumination. LED lights have 75% less power consumption and last 25% longer than traditional incandescent lamps [2]. With the decreasing prices and low power consumption of LED’s, it is estimated that the market share of LED lighting would increase to 69% in 2020 [1].

The exponential growth of data in the last two decades has raised concerns over the electricity consumption of information communication technology (ICT) infrastructure. It was estimated that ICT infrastructure accounted for 4.6% of worldwide electricity consumption in 2012 and projected to increase in the future despite emphasising on the introduction of power efficient technologies [3,4]. By 2030, the contribution of the ICT in greenhouse release would increase up to 23%, and at the worst, it can go up to 50% [5]. The future of the internet of everything (IoE) connecting people, processes, things, data and everything would require internet connectivity at all times. IoE would further increase the deployment of ICT infrastructure, thus increasing the power consumption. Apart from providing illumination at low-cost, LED lights have been used in several other applications, e.g., indoor farming and plantation [6,7], medical applications [8,9]. The easy availability of LED lights at home, offices and public spaces make it an affordable candidate to deal with the radio frequency (RF) spectrum scarcity as well as providing an energy efficient communication system. It is envisioned that existing lighting infrastructure should provide illumination as well as data connectivity.

Visible light communication (VLC) is a new paradigm that could revolutionise the future of wireless communication. In VLC, information is transmitted by modulating the visible light spectrum (400–700 nm) that is used for illumination. The information signal is superimposed on the LED light without introducing any flickering to the end user. Thus, it would be “green” as compared to providing two separate sources for illumination and communication network connectivity. On the other hand, the exhaustion of low-frequency bands to cope with the exponential growth for the highspeed wireless access is another reason for exploring new technologies. The visible light spectrum is unlicensed and hardware readily available, which can be used for data transmission. Furthermore, the exponential improvement in the high power light emitting diodes is an enabler for high data rate VLC Network. It has the potential to provide high-speed data communication with improved energy efficiency along with security/privacy. Standardisation efforts such as visible light communications association (VLCA) standards and IEEE 802.15.7 shows that VLC would augment existing wireless networks in coming years. VLC can have applications in indoor wireless communication [10], intelligent transport system [11,12], smart cities [13], localisation in warehouses/robotics [14,15,16], human sensing [17], safe and hazard-free data access in hospitals [18], toys and theme parks [19], indoor point to point (PPP) communication and vehicular communication [12].

VLC in its basic form like any other communication system in the downlink consists of a LED as a transmitter, a free space optical communication channel and a photodetector or an image sensor as a receiver. The uplink could be a WiFi transmitter or an IR transmitter or LED based VLC transmitter. Much of the work is focused on the downlink transmission to increase the data rate and improve VLC performance under different environmental conditions such as shadowing, non-LOS scenario and mobility. However, the uplink is equally essential for seamless integration of VLC in the existing ICT infrastructure. Unlike other review articles, this paper provides an overview of the VLC from the uplink and system perspective. This review article critically analyses the existing solutions of the VLC network from the system as well as uplink perspective. This paper has following contributions

- We have critically analysed the existing literature of the VLC regarding the uplink from the user device to VLC access point (VAP).
- We have discussed the open challenges associated with the use of existing RF spectrum for uplink connectivity.

The rest of the paper is organised as follow: Section 2 discusses the history and standardisation efforts. Section 3 discusses applications of VLC. Section 4 gives an overview of the different technologies used in the uplink and discusses its limitation. Section 5 summarises open research challenges toward the integration of VLC in the existing systems. Section 6 concludes the paper.

## 2. Visible Light Communication System

In the early 1990’s, mobile phones were mainly used for voice conversation or text messaging. However, the introduction of the iPhone in 2007 has started a new era in wireless communication [20]. Nowadays smartphones are equipped with all kinds of sensors and applications to provide health monitoring, video chatting, online streaming along with bank transactions and using cloud services. A larger bandwidth should be allocated for wireless communication in order to provide seamless connectivity and higher data rates. However, frequency spectrum below 5 GHz is well utilised, which leave no room to relocate spectrum for mobile communication. This led scientists to seek new wireless technologies that can fulfil the needs of the higher data rate at a low cost. One such candidate is the use of the visible light spectrum. It has the advantage of its availability (LED lights), link level security, higher bandwidth, and frequency reuse. Furthermore, the demand for data is higher in an indoor environments because 80% of the time people stay in an indoor environments [21]. Thus using the VLC in indoor applications for high data rate applications would free up the precious RF spectrum for other future applications such as autonomous cars and smart cities.

The history of VLC goes back to Romans when polish metallic plates were used to reflect sunlight and convey signals over a long distance. In 1794, Claude Chappe developed a semaphore system consisting of a series of towers equipped with mounted arms to transmit information. In the late 19th and early 20th Century, heliograph was used for long distance communication. In heliograph, the sunlight was reflected with a mirror to transmit Morse code. The British and Australian armies used it till 1960. Graham Bell is mostly known for his invention of the modern telephone, which uses electricity to transmit voice. However, Bell described the photo-phone as one of his important inventions [22]. Photophone uses voice to vibrate the mirror, which in turn is used to modulate the sunlight. It was the idea of Graham Bell, which led to fibre optic communication. The first commercial fibre optic communication system was deployed in 1975 and was capable of operating at a bit rate of 45 Mbit/s. VLC is a form of optical communication that instead of using a guided media (fibre optic) operates in the open air in close proximity of two to three meters. In 2003, the term VLC was coined first by Nakagawa Laboratory at Keio University, Japan [23]. The Nakagawa Laboratory demonstrates the first VLC system at Keio University in 2000. Light emitting diodes (LEDs) are used for transmitting the data.

Liang et al. have proposed a VLC system consisting of red-green-blue (RGB) LEDs as a transmitter and complimentary metal oxide semiconductor (CMOS) image sensor as a receiver [24]. The authors observe that RGB LEDs can increase the VLC transmission data rate with wavelength division multiplexing (WDM). For such systems, colour signals are isolated using colour filter array which relies on colour-sensitive sensing elements. However, the wide optical bandwidth of colour filters can increase spectral overlap between channels and can cause inter-channel interference (ICI). In order to solve this issue, the authors have applied CMOS image sensors with multiple input–multiple output (MIMO) to mitigate the ICI and demodulate the rolling shutter pattern.

In [25,26], Chow et al. have proposed a VLC based communication system that consists of an LED panel working as a transmitters and CMOS based camera as a receiver. In [27], Chow et al. have further improved their work using RGB LEDs to provide non-flickering for long-distance communication. The rolling shutter effect (RSE), and under-sampled modulation (USM) is analysed for VLC under different international standard organisation (ISO) values and distance. Results show that USM has better error performance over long distance compared to RSE.

Different research groups have demonstrated that a single gallium nitride (GaN)-based LED can achieve a data rate of up to 4 Gb/s [28,29]. A high data rate of up to 15 Gb/s and 13.5 G b/s using a GaN blue laser diode was achieved for a distance of 15 cm and 197 cm, respectively [30].

Zafar et al. have explored laser diodes (LDs) as an alternative to LEDs for visible light communication [31]. The use of micro LEDs can significantly improve modulation bandwidth and can provide data rates of up to 3 Gbps. However, at a high data rate, current LEDs might suffer from efficiency droop which can occur due to electron overflow. This might increase the cost of LEDs as LED will not be operating at optimum efficiency in high data rate scenarios. In contrast to LEDs, LD can offer higher direct modulation bandwidth and can provide good optical-to-electrical conversion efficiency without drooping. Therefore it is expected to provide high performance due to the characteristics of high optical power and light beam conversion. This could be used as an alternative to LEDs for illumination and data transmission. While LDs have several advantages over LEDs, LDs have many challenges of their own such as speckles, power limitations and cost that needs to be addressed. Nevertheless, VLC is still in its development phase, various features of LDs must be considered in future VLC scenarios.

Watson et al. have developed a VLC system based on GaN laser diodes aimed at unmanned underwater vehicles (UUV) [32]. The low loss of blue spectrum originating from laser makes LDs good option for underwater communications compared to traditionally used acoustic systems which are slow and proven to be susceptible to interception. Laser-based VLC systems which traditionally use non-return-to-zero on-off keying (NRZ-OOK) could achieve data rates of up to 4 Gibts/s. The authors of this study have used direct modulated GaN LD which emit light at 450 nm, and a data rate of 4.7 Gbits/s is achieved. Additionally, the underwater tracking system is developed, that, along with tracking, can provide data transmission as well.

### 2.1. Standarisation Efforts

In 2003, a VLC consortium (VLCC) was formed to speed up the research and commercialisation of VLC. The VLCC proposed two standards by 2007 [23]; JEITA CP-1221 (VLC system) and JEITA CP-1222 (VL ID system) that was later accepted by Japan electronics and information technology industries association (JEITA). CP-1223 was introduced as a VL beacon system in 2013. Both these standards have meagre data rates of up to 4.8 Kbps.

#### 2.1.1. IEEE 802.15.7

Due to increasing interest of researchers in VLC, the VLCC has introduced the first IEEE 802.15.7 standard in 2009 [33]. The standard defines the physical and media access control (MAC) layer parameters for short-range optical wireless communication. It covers topics such as network topologies, modulation domain spectrum, MAC protocol specification, collision avoidance, addressing, performance, quality indicators, dimming support, coloured status indication, and stabilisation. The standard proposes one-off keying (OOK), color shift keying (CSK) and variable pulse position modulation (VPPM) techniques for indoor and outdoor communication [34]. The highest achievable data rate for indoor communication can go up 96 Mb/s. However employing the multiple input–multiple output (MIMO) system and other modulations scheme, the data rates can be improved significantly.

#### 2.1.2. OpenVLC

Wang et al. have proposed and demonstrated an OpenVLC system, rapid prototyping, flexible and open source VLC system for the research community [35]. It provides an interface between VLC front-end with the embedded Linux platform. The hardware consists of beaglebone black board (BBB), and a transceiver front end consisting of a single LED which can serve both as the transmitter and receiver. A single LED in dual mode is used to reduce design complexity. A software-defined switch operates the transceiver. In transmitter mode (Tx) the LED is connected to the power amplifier, and in the receive mode (Rx) it is connected to a low noise amplifier. OpenVLC has implemented both the time-division duplex and IEEE 802.15.7 protocol that includes software programmability, carrier sensing, TCP/IP interoperability, encoding and decoding, preamble detection and signal sampling. The upgraded version of OpenVLC1.3 improves the data rate from UDP throughput of 100 kbps (in OpenVLC1.2) to 400 kbps without any modifications to the existing hardware [36]. It also reduces physical footprint (as it runs on off the shelf microcontroller) along with memory efficient frame detection technique. Techniques were introduced to reduce noise due to high-frequency components along with the synchronisation issues of the frame receptions. OpenVLC facilitates the research of VLC both for academia and industry.

In [37] authors have challenged the assumption that light sources are always static and users can expect LOS with many luminaries, and many scenarios together can provide deterministic localisation. The authors observe that these assumptions may not hold for many scenarios. For example, when the nodes consist of a single light and are mobile (e.g., bikes or swarms of robots) the localisation become non-deterministic. A framework is proposed to compute the relative position of objects when nodes are moving freely in all directions. The proposed framework has been implemented with OpenVLC platform. A good error rate of below 5cm is achieved through simulations.

In [38] an inexpensive receiver has been designed to cope with the issues arising due to optical noise and the mobility of users. This receiver is based on the OpenVLC platform; photodetectors are utilised to sense optical noise arising from the sun and other sources. The physical and data link layers are modified to adjust the receiver to the detected noise. Experiments were conducted for two nodes trying to maintain a link under different paths (e.g., straight and curved) and illuminations (e.g., night and day). Results validate that the noise sensing with photodetector outperforms LED only design in optical noise and mobility.

## 3. Applications

The concept of the IOE expands the network connectivity to the intelligent connection of people, data, processes, things, machine and everything. IoE would require internet connectivity for billions and trillions of sensors to provide ubiquitous, seamless services to people, machines, process, and things. All these applications would have a different set of requirements such as high data rate in Giga b/s, reliability, availability and security. The ease of availability, low cost and high data rates of VLC could make it a relevant wireless communication technology that would cater to all kind of future applications. Some of the potential applications of VLC are discussed as follow.

### 3.1. Intelligent Transport System (ITS)

**Figure 1:**
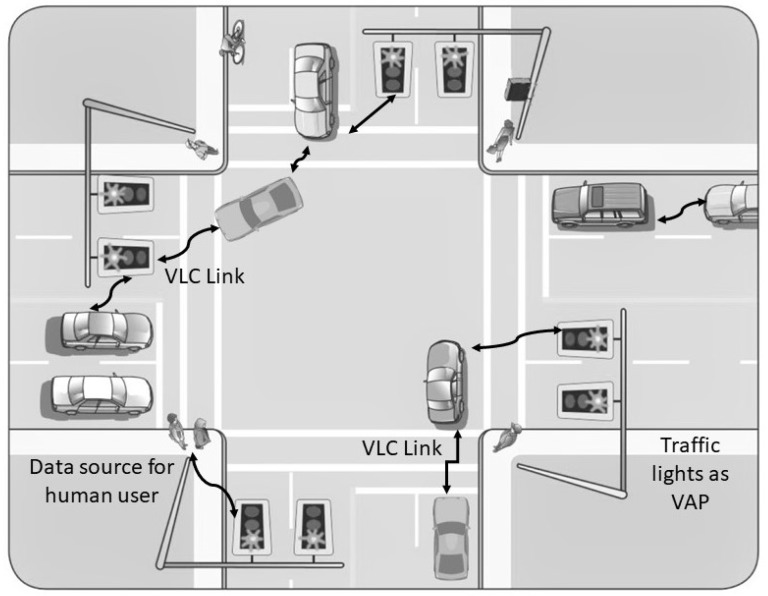
An intelligent transport system using visible light communication (VLC).

Nearly 1.2 million people die in a traffic-related incident every year, and an estimated 50 million get injured [39]. Researchers have shown that most of the incidents are due to the slow response and inability of automobile drivers to take the right action at the right time [40]. In ITS, vehicle to vehicle (V2V) and infrastructure to vehicle (I2V) communication ensures the safety of people, traffic flow and comfort of drivers as shown in Figure 1. ITS relies on reliable, robust and secure communication among vehicle and infrastructure (traffic lights, billboards). VLC is proposed for ITS communication to complement or replace the existing crowded RF-based communication [11,12]. All vehicles are equipped with head and tail lights that can be used for transmitting information. Traffic lights or billboards can also be used for sharing useful information about the road, traffic and weather conditions. These lighting sources can also be used for providing data connectivity to users and IoE entitites. Căilean et al. have discussed challenges facing VLC in the context of vehicular communication (VC) [41]. Increasing communication range, enhancing mobility and data rates are the main requirements for VC. The accomplishment of these objectives depends on the ability of communication channels to be resistant to parasitic light (PL). The outdoor channels are exposed to different kinds of PLs. It is observed that VLCs distance measuring and localisation capabilities could be beneficial in VC applications. Further, it is suggested that the development of heterogeneous systems consisting of VLC and dedicated short-range communication (DSRC) (or any other RF-based scheme) could lead to a reliable system for VCs as each of these technologies can make up for each other deficiencies. In this regard, a survey of VLC concerning 5 GHz DSRC in a hybrid arrangement is conducted. It is concluded that VLC systems aimed at VCs can be improved by exploring and integrating new technologies which include but are not limited to software-defined architecture, resource sharing, reconfigurable computing and integration of new materials. Ucar et al. have developed a hybrid 802.11p and VLC secure autonomous platoon system [42]. The autonomous platoon uses RF based 802.11p and consists of a platoon leader that controls other members to adjust the speeds stably. A 802.11p and VLC hybrid vehicular platoon communication protocol, named as SP-VLC is proposed. This protocol is aimed at addressing security vulnerabilities due to the exclusive use of RF communication. A simulation platform for the vehicle mobility and vehicle platoon managementis is developed. SP-VLC is evaluated under different security vulnerability scenarios. The simulation results validated the observations made in [41] that RF-VLC heterogeneous systems can provide many advantages over the RF only system.

Kunar et al. have proposed to integrate LED-based road side units (RSU) into the existing ITS infrastructure [43]. The RSUs are used to broadcast information in infrastructure to vehicle (I2V) mode using VLC concepts. A robust modulation technique based on a direct sequence spread spectrum (DSSS) and sequence inverse keying (SIK) is employed to minimise the effect of noise sources. The amount of data received by a car passing by RSU is considered as a performance metric. The experimental setup involves a movable receiver and a stationary emitter both separated by a distance of 1.5 m. Results show that packet error rate (PER) degrades linearly with distance during daylight while at night the packet error varies due to the local nature of artificial light.

In [44] performance evaluation of VLC based V2V systems has been conducted. For performance evaluation, a typical V2V VLC scenario is considered with left and right headlamps emitting light. The reflected rays are considered to have both line of sight (LOS) and non-line of sight (LOS) components with the Lambertian profile. Results show that depending on the headlamp location a data rate of 50 Mbps can be achieved at a separation distance of 70m between vehicles.

In [45] the authors have considered VLC-based ITS for accident avoidance, particularly, when lorry fleet are moving through intersections. VLC has been used to send signals related to acceleration, deacceleration and braking to ITS infrastructure (e.g., RSUs) which can trigger appropriate signal. For example, to reduce the number of emergency brakes and lane change in a complex environment, a lorry fleet can send VLC signal to RSU which can set a green signal or express path.

Yamazato et al. have used VLC with imaging sensor-based receiver for automotive applications [46]. Two scenarios I2V and V2V are considered. The first scenario consists of a transmitter designed from LED arrays (assumed to be RSUs) while the receiver is considered to be high frame rate CMOS imaging camera. In the second scenarios, a special CMOS sensor have been developed which can receive high-speed optical signals. In the field trials, a data rate of 32 kb/s and 10 Mb/s is achieved for I2V and V2V, respectively.

### 3.2. Smart Cities and Smart Homes

Smart cities are envisioned to provide seamless connectivity between people, government, infrastructure, economy and environment [13,47]. Most of the functional entities of a smart city are already available around us. However, the reliable, sustainable and high data rate wireless connectivity is the bottleneck to connect all the enablers. The already available infrastructure of lightning (street lights, parking lights, billboards) can be utilised to provide high-speed, low energy and sustainable network connectivity for some applications (e.g., utility services) in smart cities whereas freeing up the precious RF spectrum for other mobile applications. Street lights or other lighting sources could be used as a hotspot to provide extremely high data rates to the user.

A three-layer VLC based communication architecture is proposed to integrate different technologies in smart cities’ applications seamlessly [48]. Layer one uses VLC to allow user access and a sense of events. Layer two provides communication between different LEDs and sub gateways. The last layer provides communication between different sub gateways and the service gateway using optical communication. Based on this architecture several applications (such as intelligent communication, event surveillance, and object tracking) have been demonstrated. In [49], authors have investigated optimisation algorithms to provide consistent received optical power and signal to noise ratio (SNR) across a single receiver plane in smart homes.

In [50] a hardware design and location identification protocol are demonstrated for a VLC-based indoor positioning system for smart supermarkets. The hardware design considers the illumination flickering, brightness, signal synchronisation and noise in the indoor environment. Various practical user conditions have been used to optimise the hardware and software implementation in terms of location accuracy and bit error performance. It is concluded from the experimental work that location ID detectability is 95% for a distance of 0.7 s and under the illumination in diameter of 2 m.

### 3.3. Entertainment and Advertisement Industry

VLC requires line of sight (LoS) or semi line of sight. This can be used for applications requiring access to localise information. Theme parks, toy or advertisements are such applications, where the aim is to produce products with reducing costs. For example, most of the toys are battery powered and operates in LoS, and typically require a wireless connection to control the toy [51]. Some researchers have explored battery-free VLC communication system. Xu et al. have developed a new VLC based communication, called passive VLC that uses the backscatter communication for battery-free IoT applications [52]. This system is based on RetroVLC which tackles the challenges of directional communication and low optical coupling (mostly less than 20% via solar panel) for wideband VLC. RetroVLC uses the concept of retroflecting which involves sending light back, almost, along with the incoming path, achieving a pointed communication. Miller codes are used to double bandwidth utilisation compared to Manchester coding. Trend-based modulation and code-assisted demodulation are proposed for reducing the modulation time. Two proof of concept systems are developed to validate their proposed scheme. A data rate of 1Kbps was achieved with the RetroVLC system.

A single LED light can be used for transmission and reception as well as providing the visualisation to entertain kids. This would save energy and cost, also not polluting the precious RF spectrum. Another use of VLC could be in the advertising industry, where large billboards made of LED’s are used for advertisement. Such large billboards with high light intensity and equipped with hundreds of LED ’s can be used for providing free outdoor network connectivity that can work as hotspots for users, machines or smart cities applications.

### 3.4. Hospitals

VLC operates in 430–770 THz, and due to nanometer wavelength, it cannot penetrate in objects, therefore making it ideal for applications where confidentiality of data is of utmost importance. The inherent security and safety feature of VLC provides an alternate of wireless communication that could be used to minimise health risks associated with radio frequency radiations. One such area is hospitals, where VLC can be utilised for monitoring patients, machine to machine communication, record keeping of patients and all other networking applications [53]. Newborn babies are fragile and vulnerable to even small RF radiations of the ISM band and cellular frequencies. Implementation of VLC in newborn babies wards would be a natural choice for monitoring and indoor connectivity to the rest of the internal and external networks. To replace the existing wireless communication in hospitals, a broadband powerline and VLC with orthogonal frequency division multiplexing (OFDM) is demonstrated [53]. Abdaoui et al. have proposed another hybrid system consisting of VLC and 60 GHz RF for e-health applications [54]. The rationale of VLC for these applications as it is easily available in hospitals and can reduce communication system cost. As VLC offers only downlink communication, therefore, 60 GHz RF to provide uplink communication is used. Apart from uplink, 60 GHz can be used for localisation as well. It is concluded that this hybrid system could be used efficiently to collect a wide range of information from the patients.

Chow et al. [25] have demonstrated secure data transmission that can have applications in a hospital environment. In [25] authors have proposed a CMOS camera-based VLC system which encrypts emitted light. The transmitter consists of an encryption module which receives an optical signal that is encrypted using a private key or another advanced encryption scheme and is then emitted. The CMOS-based mobile camera is used as a receiver. The rolling shutter feature of the CMOS camera is used to enhance data rates. An additional Otsu thresholding module is used to get effective BER. The proposed scheme gives good results in terms of BER and encryption.

A communication system inspired by barcoding technology is proposed [55]. Unlike VLC, this system considers that without explicitly modulating a light source, the disturbances in the natural (e.g., sun) can be used to convey information and thus it is named as a passive communication system. It involves the design of a communication channel in which information is captured from the mobile elements with wear patterns consisting of distinctive surfaces. A user can be reduced to a “tiny-box” equipped with a commercial off-the-shelf (COTS) photodiode. Massive deployment of these tiny boxes can infer significant pieces of information from the surrounding. Such systems are deployed in hospitals where emergency, treatment and housing trolleys can report their physical locations. A key aspect of this system is sustainability in terms of infrastructure (as it needs of minimum infrastructure changes), energy efficiency (as the system uses only photodetectors which are energy efficient compared to the camera used in similar scenarios) and cost (as photodetectors are inexpensive). The passive communication system is demonstrated in the parking area and reported encouraging results.

## 4. Hybrid VLC

**Figure 2:**
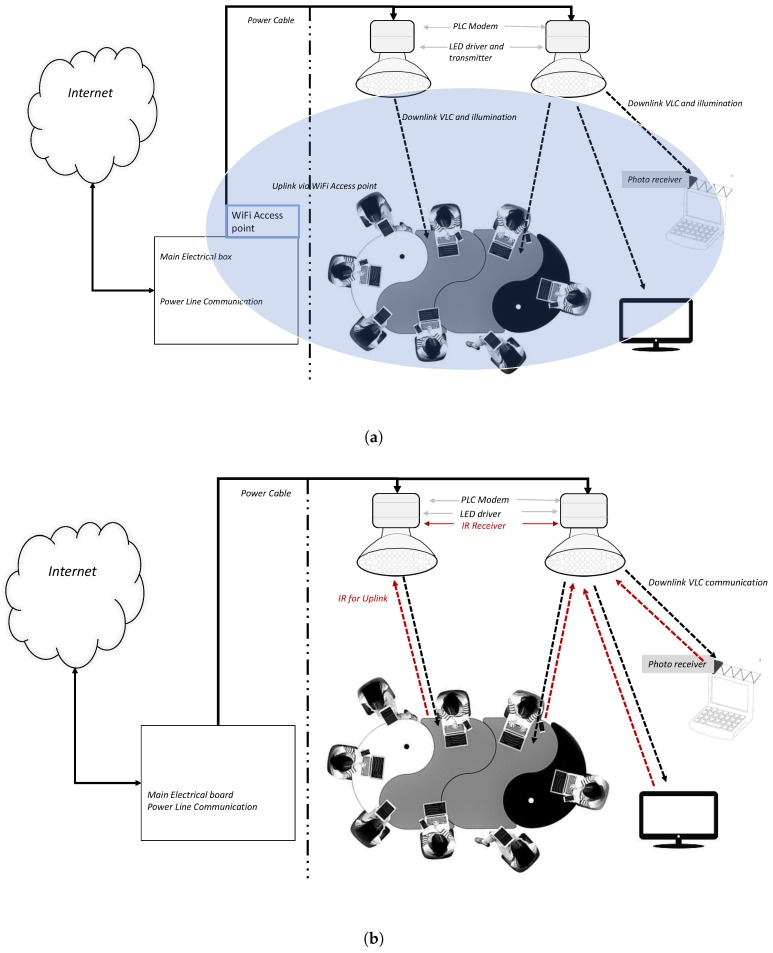
VLC downlink and infrared is used for uplink in VLC Hetnet. (**a**) VLC downlink and WiFi for uplink in VLC Hetnet. (**b**) VLC–VLC communication.

A VLC network arrangement is shown in Figure 2a. The downlink consists of a LED driver which drives the LED illuminator to work as a VLC transmitter and a power line modem to connect the VLC transmitter to the network. For simplification, this downlink VLC transmitter is called a VLC access point (VAP). The power line communication (PLC) technology is typically utilised to connect all the VAPs to the rest of the network both internally and externally. In the user devices, the downlink VLC transmission can be received either through a photodiode or an image sensor. The uplink from the user device could be through either (a) VLC transmitter, (b) a wireless fidelity (WiFi) link, (c) an infrared (IR) link, (d) a combination of any two. Figure 2b shows another arrangement of the VLC network. An IR transmitter is used on the uplink instead of a WiFi. In this scenario, VAP consists of PLC modem, VLC transmitter, and an infrared receiver (IR). The user device consists of a photodiode to receive the downlink VLC transmission and an IR transmitter to communicate on the uplink. Both IR and VLC require line of sight for communication. Instead of an IR transmitter, we could use a LED transmitter for the uplink to make a bidirectional VLC link. However, as light would be continuously emitting in an upward direction, which is aesthetically not pleasing from a user perspective.

In any case, the VLC would occur through a heterogeneous network that would be using more than one networking standard, i.e., a combination of PLC-VLC, PLC-VLC-WiFi, PLC-VLC-IR. There are limitations of each existing technology to support VAP. For example, PLC is used to connect the VAP with the rest of the network. PLC can achieve a data rate of 1 Gbit/s [56,57]. Thus creating a bottleneck to use VLC for high data rates although theoretically, VLC can provide high-speed data up to 15 Gbit/s [30]. Some researchers have considered fibre optic as an alternative to PLC [58]. However, it will increase infrastructure cost.

**Figure 3:**
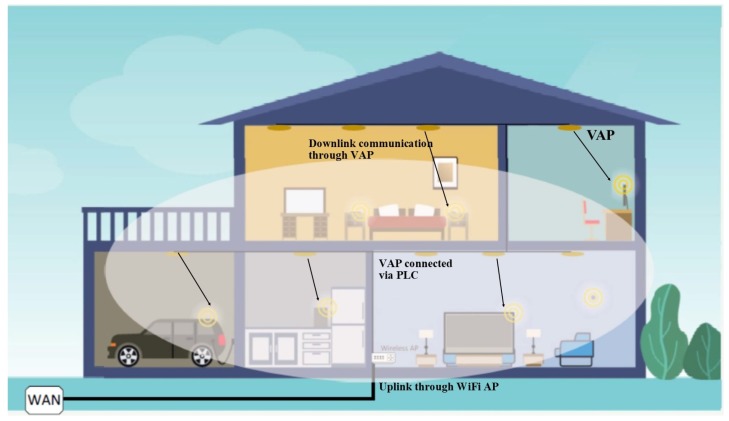
WiFi VLC communication network.

Figure 3 shows a typical deployment of VLC network in a house. It consists of a heterogeneous network of PLC-VLC-WiFi. One WiFi AP is used to cover the entire house whereas VAP’s are distributed throughout the house and connected via PLC to the main gateway. This arrangement is cost effective and realistic as typically the links are asymmetric considering that uplink can have low data rates as compared to downlink transmission. VLC users at the boundary of WiFi would have high signal attenuation, which could deteriorate the overall throughput. A second arrangement is to deploy low power WiFi transmitters (WiFi Direct) in VAPs. However, it would increase the complexity of hardware and software in VAP. Furthermore, VAP’s are prone to interference as it cannot penetrate objects due to characteristics of its frequency. However, WiFi operates in 2.4 and 5 GHz range, and it can penetrate in walls. Therefore fine tuning WiFi transmitter to a very small area would be practically unachievable. This will increase the interference among wifi transmitters in close proximity. A third alternative is to deploy multiple WiFi to provide good coverage and throughput, but it would require excellent RF planning to mitigate interference issues. We will discuss this in detail later in the next subsection.

In most of the cases, photodetectors are employed as a receiver for the VLC. However CMOS sensor-based camera are also experimented with and demonstrated for reception of visible light and extracting information from it. Danakis et al. have developed a proof of concept system which shows that rolling shutter feature of CMOS sensors can be used to achieve higher data rates [59]. The overall system consists of an LED transmitter, a mobile camera-based receiver, and an Android application equipped with a decoder. The information is captured in a mobile phone as a light and dark band which can be decoded by mobile phones to retrieve information.

### 4.1. WiFi-VLC

WiFi (wireless fidelity) is a trademark of WiFi Alliance, based on the 802.11x family of Ethernet standard for wireless local area networks (WLANs). Devices can connect to the internet via a WLAN access points (APs). WiFi is primarily used for internet access in the indoor environment connecting via wire to the backbone network [60] as shown in Figure 3. WiFi direct is another variant of WiFi, providing direct connectivity between user devices without any intermediate device [61].

The main application of VLC is to provide high-speed connectivity in an indoor environment. Light fidelity (LiFi) is one such indoor application of VLC, proposed by Herald Hass in 2011 [62]. LiFi promises a fully networked indoor system with bidirectional support for point-to-point (P2P) and multipoint-to-point communication (MP2P). It also provides the capability of multi-access points and the formation of wireless networks between them resulting in LiFi atto cells (comparable to femtocells). These atto cells provide seamless handover and promise to provide full user mobility. Different researchers have proposed different system models for LiFi [63,64,65,66]. One of the proposed models is to augment LiFi with WiFi, where the latter works as an overlay network over on the former. In another approach, a mixed WiFi and LiFi is used to provide an aggregated system providing aggregated channels for communication. Light radio (LiRa) is another application developed by augmenting VLC with legacy WiFi [64]. LiRa does not require VLC for the uplink transmission by the mobile client and instead employs a WiFi uplink. The system achieves VLC automatic repeat request (ARQ) feedback via WiFi without excessive delay with a newly introduced method, AP-spoofed multi-client ARQ (ASMA). This method provides the mechanism to minimise the impact of VLC control frames on legacy WiFi traffic while providing enough feedback for the visible light downlink.

Khreishah et al. [66] proposed a VLC-WiFi hybrid system for energy efficiency. An analytical framework for energy efficiency is proposed that is based on two requirements; (a) selection of the appropriate number of APs to provide the lighting, (b) to satisfy the user’s request for real-time communication. The results showed that the VLC-WiFi hybrid system is 75% more efficient than a standalone WiFi network. Kashef et al. carried out a similar investigation for energy efficiency and LOS availability of a VLC-RF hybrid system [67]. The system parameters that affect the energy footprint of the communication system are analysed. Results showed that energy efficiency improved at the cost of less LOS availability.

Shao et al. have proposed two variants of hybrid systems as proof of concept for the coexistence of WiFi and VLC [68]. In the first phase, a WiFi-VLC hybrid system is proposed where VLC is used for the downlink and WiFi for the uplink transmission. Results have shown that higher data rates can be achieved compared to the conventional WiFi systems. In order to take advantage of the higher bandwidth of VLC on the downlink and exploit the higher availability and range of WiFi, the hybrid systems are further enhanced by bundling together WiFi and VLC channels using bonding techniques based on the concept used in the Linux operating system. Practical experiments are performed in the crowded environments for comparing the hybrid system with WiFi only system. Robustness and throughput are used as performance metrics. Results have shown that hybrid systems are robust and provide higher throughput compared to WiFi systems.

In [69], a hybrid system has been developed which consists of optical femtocells overlaid over WiFi cells, named as WiFO. The WiFO exploits the high bandwidth of optical femtocells and high mobility of WiFi to provide high throughput along with seamless handover and good bit error rate (BER). This system involves, among other components, a custom network protocol with physical, link, network and transport layers, to support the developed system. While other layers are similar to traditional networking layers, the networking layer, however, is modified significantly to provide seamless mobility in addition to routing packets. The authors have performed experiments and have shown that the optical femtocells in the WiFO could achieve a data rate of up to 50 Mbps at a distance of 3 m.

In [70], a hybrid VLC and RF system is considered that exploits the longer range of RF to provide control functions and increase per user rate coverage of the VLC cells. It quantifies the minimum spectrum and power requirements which can achieve a certain per user outage performance. The hybrid system consists of central unit (CU) which assign users to either VLC or RF network based on the user’s channel conditions (users with the low data rate in the VLC network are migrated to RF network). The proposed system is evaluated for two scenarios, D1 and D2. In D1 power and spectrum are kept constant while in D2, CU dynamically adjusts power and spectrum according to the traffic demands. Results have shown that that D2 outperforms D1.

In [71] a hybrid VLC and RF system has been proposed which aims to support diverse quality of service (QoS) needs of users. The QoS needs are studied for limits on the buffer overflow and delay violation probabilities. Results show that RF performs better for arrival rate in some settings, while VLC has better delay performance.

In [72] a hybrid WiFi-VLC system has been implemented which consists of two duplex links. One is an aggregate of VLC and WiFi, and the other is WiFi duplex link. WiFi is used for the uplink transmission. The Linux bonding technique is used to achieve link layer aggregation using the Linux bonding driver to build a logical interface (LI) on the client. Throughput and round-trip time (RTT) of the hybrid systems are evaluated under different contention scenario and VLC operating distances. The proposed system was benchmarked against the conventional WiFi and a hybrid WiFi-VLC system (non-aggregate system with asymmetric VLC downlink and WiFi uplink). Experiments have shown that the throughput of the aggregate system outperforms the standalone WiFi and a conventional WiFi-VLC hybrid system. The RTT of the aggregate system shows considerable improvement over the benchmark system particularly when the WiFi is facing congestions.

Most of the existing work in VLC-WiFi hybrid system is based on a single VAP in a confined area. WiFi is used for uplink in close proximity similar to WiFi direct. As shown in Figure 3, one WiFi AP can cover multiple VAP’s. However, WiFi signals attenuate over the distance, which would increase the errors in the uplink and thus feedbacking in the downlink to decrease the data rate. As the VAP is deployed at multiple locations to cover a large indoor area, then the collaboration between different VAP and one or more WiFi is not evaluated. This creates a bottleneck towards the scalability of VLC-WiFi network. Furthermore, there has been less reported work on the cost, fault tolerance, security and overall throughput of the systems.

### 4.2. IR-VLC

Most of the current research relies on IR or RF for uplink transmission [73]. Infrared (IR) indicates the light with a wavelength less than red light. It has a frequency range between 300 GHz and 430 THz. IR band is unlicensed and has the potential to provide high-speed connectivity. Since the frequency is above 300 GHz, it cannot penetrate objects, hence used mostly in the indoor LOS environment. According to Boucouvalas et al. [65], IR can achieve up to 1 Gbits/s of data rate over a distance of several meters. IR communication can take place in defused mode, also called scatter mode. In this type of communication transmitter and receiver do not need to be in the line of sight (LOS). However, for such communications devices need to be close to each other which can be an issue when used for developing indoor wireless networks. Researchers are now taking particular interests in using IR to complement VLC network in the uplink transmission.

Alresheedi et al. [74] have proposed fast adaptive beam steering IR (FABS-IR) system to enhance the received optical power signal, speed up the adaptation process and reduce the channel delay during high data rate operation. The proposed scheme employs IR for uplink transmission and VLC for the downlink transmission. FABS-IR was coupled with imaging receivers to improve the system performance. Results showed that employing the IR-VLC network increases the data rate up to 2.5 Gb/s for a mobile scenario in an indoor environment. Quintana et al. [75] have proposed a VLC-IR system for internet access to passengers on flights. The VLC-IR system can also be used to provide personalised entertainment using wireless media without interfering with airline radio systems. The proposed system claims to have minimal interference as every lamp serves as VAP dedicated to each seat. Experiments have shown significant minimisation in transmission error for a short distance (2 m), good downlink signal strength for receivers, and functional capability to work in the presence of other illumination sources. Further, the proposed system is tested for other onboard entertainment such as collaborative games and live video streaming.

Few researchers have investigated IR for uplink transmission in the VLC as compared to WiFi. WiFi is already available, and it has hardware and software support available, whereas integrating IR in experiments are difficult due to less support of hardware and software. IR has less data rate and a lower range of communication, which made it less attractive for the researcher to investigate. Furthermore, IR transceiver has to be integrated into the VAP and in the user device, which further increases the complexity of the VLC system. Nevertheless, researcher community has shown a high interest in improving the IR-VLC in an indoor communication system for specific applications such as in-flight communication system, point-to-point communication. IR communication could be one of the areas, which would complement VLC either in the uplink or during mobility.

### 4.3. PLC-VLC

For VLC to provide a fully functional indoor network, it requires connectivity among VAPs and external networks. The easy and straightforward solution is to connect VAP via separate cables. However, this will increase the cost of cabling, installation, labour and design. PLC utilises the already installed electrical power lines for broadband access [76]. PLC modems are typically plugged into power sockets to provide the interface between data and power network. For over a decade, PLC has been successfully used to transmit radio programs, home automation (remote control of lighting and appliances), home networking, internet access, utility company control switching and automated meter reading [77,78]. Thus like VLC, PLC has the advantage of having the readily available infrastructure.

Hu et al. [79] proposed PLiFi, a Hybrid WiFi-VLC that promises low-cost connectivity to the internet, interconnectivity between the VAPs and seamless integration of WiFi for the uplink transmission. The user mobility and change in device orientation is investigated. A smaller and distributed VAP along with a MAC protocol coordinated multi-point transmission (CoMP) is proposed to address the co-channel interference due to adjacent LEDs. In the experimental setup, PLC modems were used to synchronise VAPs.

Ndjiongue et al. [80] have developed a cascaded PLC and VLC channel model of hybrid PLC-VLC systems. The influence of PLC channel and VLC transmitters on the propagation of the signal is analysed. The available channel frequency response is used to determine the transfer function to characterise the channels over time. The cascaded channel path of PLC-VLC can have many sources of impairments including but not limited to attenuation and impulsive noise. The proposed model claims to analyse the cascaded channel attenuations accurately. However, noise is ignored in their analysis which can be a source of impairment in the PLC-VLC environment.

Yan et al. [81] have performed experiments with three-LED lamps to form a single frequency network (SFN) that showed the feasibility of VLC and PLC hybrid system for the indoor environment. Their system promises high-speed communication and greater signal coverage with less modification to the existing infrastructure. A similar experiment is performed by song et al. [82]. They have implemented a full duplex decode and forward (DF) hybrid PLC-VLC system with minimal protocols or medium modifications. A data rate of 5 Mbps was demonstrated with their experimental test bed, which can be extended theoretically up to 30 Mbps.

The research work as mentioned above provides some theoretical models as well as some practical implementations for setting up a PLC-VLC hybrid system. It provides an investigation into some essential components of hybrid systems such as channel modelling, noise removal, interference mitigation, and basic networking. However, hybrid systems are not evaluated for their benefits such as energy efficiency and scalability. The cost of electrical devices (e.g., PLC modems) is much higher compared to VAP, and the effect of adding these devices to the system have been ignored. According to Tonello et al. [57] data rates of up to 1 GB are possible by careful analysis of the PLC channel and appropriate selection of modulation schemes. For the current data requirements, 1 Gbps would suffice. However, VLC is capable of providing a data rate of up to 15 Gb/s [30], which is far higher than PLC. Thus, it would lead to a bottleneck. Future network application would require a backbone network that can support higher data rates. A viable solution might be to connect VAP through fibre optic, which will increase the system cost. Such VAP would potentially provide an ultra-high data rate for specific applications, where a user wants an ultra-high data rate.

In [83] optical fibre link has been used to connect central station (CS) to radio access points (RAP) for indoor applications. The system consists of an RF front-end which includes among other components, an antenna operating in 2.4 GHz, a power amplifier and a photodiode (as a receiver).

In [84] Gomez et al. have designed a fibre-wireless-fibre (FWF) link for indoor optical wireless communications. Through simulation data rates of 416 and 208 Gb/s can be achieved with a wide field of view of 40 and 60 degrees, respectively. Although these data rates are for the fibre optic communication to avoid the signal conversation from optic to electrical and vice versa. However, the same concept can be extended to the backbone access network of the VLC network to remove the bottle neck of PLC network.

### 4.4. LED-to-LED System

A typical VLC system uses LED as transmitter and photodetector as a receiver. Most of the research work has experimented with one-way VLC system in the downlink direction. To take full advantage of the large unregulated bandwidth of VLC, ideally, a bidirectional VLC system should be used for downlink and uplink communication. To build a two-way VLC-VLC system, an LED transmitter and photodetector would be required at both ends for downlink and uplink transmission. However, because of user device sensitivity to orientation changes and aesthetically not pleasing, it has not attracted much research for indoor wireless communication.

Schmid et al. [85,86] have developed a LED-to-LED VLC system based on the concept of Dietz et al. [87]. The VLC system is based on a single LED, which can be used both for transmission and reception. The discharging characteristic of LED was used for receiving the incoming light, which in turn translates to information. The LED capacitance changes with the intensity of receiving light. A threshold was set based on the experiments to determine the discharging for the different intensity of light to infer the incoming symbols of ON and OFF. In another reported work, smart phone was used to relay the VLC to a toy car [88]. The obvious drawback is that LED is less sensitive to light. A high-intensity light would be needed which will increase the cost and decrease the communication range. Also, a single LED for transmission and reception means that it would require multiplexing. Furthermore, fine tuning would be required to keep the LED illuminated without transmitting any useful information. This would decrease the maximum achievable data rate.

Wang et al. [58] have proposed a full fibre integrated LED-to-LED VLC system. A fiber optic link is used to connect all VAP’s to address the backbone bottleneck. A hybrid access protocol is designed using frequency division multiplexing for the uplink and downlink in fiber transmission, and time division multiplexing for bidirectional VLC transmission. Total system throughput of 8 Gb/s was achieved for eight VAP; each offered a 500 Mbps downlink and uplink transmission. The same network architecture can be extended to support upto 100 Gb/s wireless access system in the future.

Li et al. [89] have proposed a system which uses LED enabled devices to communicate on a two-way VLC channel without the use of an exclusive photodetector, while simultaneously providing illumination. Manchester coding with on/off keying (OOK) modulation is employed to achieve the best tradeoff between illumination and data rates. A prototype is developed to achieve data rates in the order of kbps at a distance of ten of centimetres.

Yeh et al. have used 682 nm visible-vertical-cavity surface-emitting laser (VCSEL) for the bidirectional VLC system [90]. The downstream of the proposed system is based on orthogonal frequency division multiplexing (OFDM) and quadrature amplitude modulation (QAM) modulation. To further enhance bandwidth efficiency bit-loading and power-loading algorithms are used. Results show that a single VCSEL bidirectional-VLC system can achieve 10.5 Gbits/s OFDM downstream traffic and 2 Mbits/s upstream.

Heydariaan et al. have investigated the VLC channel at the application level under different settings and scenario for low cost embedded VLC devices [91]. It investigates the effect of platform parameters such as transmitter and receiver position, the nature of receiver (whether the receiver is PD or LED-based), and the protocol level parameters such as symbol rate and frame payload size. The experimental setup involves adopting OpenVLC 1.0 with BeagleBone board and running a Debian Linux. The parameters investigated are throughput and packet reception ratio. Experiments show that channel conditions change for different scenarios and parameters need to be tuned for different scenarios such as day condition, mobility etc. The usage of a simple PD could reduce the impact of ambient light on the overall throughput.

Wang et al. [92] have developed a carrier sensing multiple access/collision detection and hidden avoidance (CSMA/CD-H) communication protocol, which provides reliable transmission for VLC networks consisting of nodes with different Field of View (FOV). Hidden node problem arises due to diverse FOVs of LEDs. CSMA/CA-H protocol enables intra frame bidirectional communication in the LED network. The proposed protocol is implemented using an OpenVLC platform consisting of beagleBone Black (BBB) board and a front-end transceiver. Experiments show that the CSMA/CD-H protocol can reduce hidden node collisions significantly while increasing throughput by 50% and 100% for two and four nodes networks respectively.

Giustiniano et al. [93] have addressed the issues of flickers elimination and efficient collision-free medium access control in bidirectional LED-to-LED networks. The system also consists of a method to measure the incoming light during data reception and transmission. Research showed that LED sensitivity, physical rate and flicker elimination are tightly correlated and lead to a tradeoff scenario. The proposed system is evaluated experimentally and shown that it can address flickers eliminations and collision-free communication with a throughput of 780 b/s.

The prevailing assumption in the VLC community is that LEDs emit constant light and can provide flicker-free constant throughput. Many of the current deployed LEDs are smart, dynamically adjusting emitted light to ambient conditions. Dynamic change can lead to fluctuating throughput and flickering, resulting in bad user experience and awkward lighting scenarios. Wu et al. have proposed a smartVLC system based upon the investigation of the trade-off between the fine-grained dimming level and high data rate throughput [94]. In order to achieve this trade-off, a new modulation scheme adaptive multiple pulse position modulation (AMPPM) is proposed which multiplexes individual symbols to achieve dimming granularity. Experiments show that an inexpensive smartVLC system provides flicker-free communication up to a distance of 3.5 m with improved throughput.

These studies have led to the development of very low-cost LED-to-LED VLC systems which can be readily applied is providing connectivity on the very low scale and cater to some specific applications. However, the reliability, scalability and robustness of such VLC based communication networks regarding handling interference, inter/intra LED-to-LED VLC communication and its energy efficiency are still less explored. Nevertheless, a LED-to-LED VLC system would be the end goal to tap into the large chunk of unregulated visible light spectrum. Some point to point applications would be potential applications for a full functional LED-to-LED VLC communications such as vehicle to vehicle and vehicle to infrastructure communication.

## 5. Discussion and Open Research Challenges

There has been increasing innovation in solid-state lighting which has resulted in the availability of efficient and low-cost devices available in the form of LED bulbs. This LED-based lighting infrastructure is now increasingly being used in indoor environments such as homes, offices and shopping malls [63], and in the outdoor environment such as for street and roads lighting [95]. Moreover, it has been shown that LED-based communication (which has been standardised by IEEE as 802.15.7) [33,96] offers higher data rates. According to some researchers, theoretical data rates for LED-based lighting communication is more than 15 Gbps [30]. While this inherent data communication capability is available on the downlink because of the readily available lighting infrastructure and user devices, equipped with photoreceivers and cameras, capable of detecting light. However, using VLC for the uplink has some issues. First, most modern devices do not have the transmitter for VLC and providing new ones will not only add to cost but also increase the device size. Second, using VLC for the uplink results in unusual lighting conditions from a user perspective which are aesthetically not pleasing. To provide seamless integration of VLC, researchers have focused on developing a hybrid system for seamless integration of VLC. In such systems, VLC is used for the downlink transmission and RF or IR for the uplink. Unlike VLC, IR and RF are already supported by most of the current networking devices. RF has a wider coverage range compared to VLC though susceptible to interference. Radio waves can penetrate objects and as a result can be a better substitute for the uplink transmission.

VLC is in the research and development phase. There are many research gaps need to be addressed before its commercial availability for home and enterprise users. Some of the research gaps is discussed as follow.

### 5.1. Inter-Cell Interference Mitigation

The small radius of a VAP cell provides higher capacity at the expense of dense deployments. Managing inter and intracell interference is a challenging task in a small and large indoor area, which is equivalent to densification of the cellular network to increase capacity. Visible light spectrum cannot penetrate objects and provides less interference out of its coverage area. However, the VAPs in the same area would cause interference leading to low signal to interference and noise ratio (SINR) and would degrade performance [97]. Beysens et al. have exploited dense deployment of LEDs and massive MIMO to propose a system, called DenseVLC [98]. It provides a battery-free VLC networking system by forming different beam spots to serve multiple users simultaneously. Optimisation techniques are used for allocating power among LEDs. DenseVLC prototype consists of 36 Tx and 4 Rx, developed from the commercial Off-the-shelf (COTS) devices and powered with the open source OpenVLC platform. The DenseVLC improves system throughput by 45% and the average power efficiency by 2.3 times in comparison with the existing techniques. It also significantly reduces inter-cell interference.

Network MIMO, joint transmission and VAP rearrangement are proposed as potential solutions for interference mitigation. In network MIMO and joint transmission, the interfering VAPs can coordinate their transmissions via interference nulling or synchronisation to ensure high SINR at the receivers. Also, interference could be decreased by rearranging the VAPs, which would be similar to cellular planning. However, the systematic design and analysis for optimising the communication performance under the illumination constraints is still an open research challenge which requires further investigation.

### 5.2. Backbone Network Design

To provide broadband access, all VAPs should be connected to the rest of the world. It would be a challenging problem to connect all the VAPs to internet considering the dense deployment for illumination purposes. Creating a new wired network of VAP with Ethernet or fibre is not a cost-effective solution. Wireless connectivity or power line communication could be a potential solution. However, it has limitations such as interference of wireless connections, the low data rate of PLC, the additional cost of integrating with VLC. A potential solution could be to free up the ISM band (2.4 and 5 GHz) providing approximately 200 MHz of a band that could be used to provide backbone connectivity to VAP.

### 5.3. Uplink Design

Most of the research in the VLC is focused on the downlink transmission to increase the data rate or mitigate interference issues. Less attention is given to uplink transmission from the user device. As mentioned earlier, a limiting factor in using the LED for the uplink from a mobile device is that it consumes not only significant energy but also causes visual disturbance to the user. Researchers propose RF and infrared. However, it would create heterogeneous networks due to different technologies at uplink and downlink. Coordination among multi-home clients, resource allocations and performance issues due to asymmetry heterogeneous networks are in the early phase of research.

## 6. Conclusions

In this article, we have discussed the visible light communication network, its history and applications. The existing literature is reviewed for the uplink transmission. Currently, most of the research work is focused on the downlink to present a strong argument to the research community about the potential of VLC regarding applications and high data rate. Most of the existing research have used RF and IR for the uplink. PLC is mostly used as a backbone in the access network. We highlighted the limitation of these uplink technologies. In order to efficiently utilise the vast bandwidth of visible light spectrum, a complete system-level evaluation is required. This evaluation should consider the limitation due to uplink and backbone. A scalable, reliable and high data rate VLC network would undoubtedly be a heterogeneous network. This article would facilitate the research community toward the system level analysis and implementation of the VLC system.
